# SETDB1 deletion causes DNA demethylation and upregulation of multiple zinc-finger genes

**DOI:** 10.1007/s11033-024-09703-2

**Published:** 2024-06-21

**Authors:** Yong-Kook Kang, Jaemin Eom, Byungkuk Min, Jung Sun Park

**Affiliations:** 1 Development and Differentiation Research Center, Aging Convergence Research Center (ACRC), Korea Research Institute of Bioscience Biotechnology (KRIBB), 125 Gwahak-ro, Yuseong-gu, Daejeon, 34141 South Korea; 2 Department of Functional Genomics, University of Science and Technology (UST), 217 Gajeong-ro, Yuseong-gu, Daejeon, 34113 South Korea

**Keywords:** SETDB1, Zinc-finger protein, DNA methylation, Hypomethylation, Hypermethylation

## Abstract

**Background:**

SETDB1 (SET domain bifurcated-1) is a histone H3-lysine 9 (H3K9)-specific methyltransferase that mediates heterochromatin formation and repression of target genes. Despite the assumed functional link between DNA methylation and SETDB1-mediated H3K9 trimethylations, several studies have shown that SETDB1 operates autonomously of DNA methylation in a region- and cell-specific manner. This study analyzes *SETDB1*-null HAP1 cells through a linked methylome and transcriptome analysis, intending to explore genes controlled by SETDB1-involved DNA methylation.

**Methods and results:**

We investigated SETDB1-mediated regulation of DNA methylation and gene transcription in human HAP1 cells using reduced-representation bisulfite sequencing (RRBS) and RNA sequencing. While two-thirds of differentially methylated CpGs (DMCs) in genic regions were hypomethylated in SETDB1-null cells, we detected a plethora of C2H2-type zinc-finger protein genes (C2H2-ZFP, 223 of 749) among the DMC-associated genes. Most C2H2-ZFPs with DMCs in their promoters were found hypomethylated in SETDB1-KO cells, while other non-ZFP genes with promoter DMCs were not. These C2H2-ZFPs with DMCs in their promoters were significantly upregulated in SETDB1-KO cells. Similarly, C2H2-ZFP genes were upregulated in *SETDB1*-null 293T cells, suggesting that SETDB1’s function in ZFP gene repression is widespread. There are several C2H2-ZFP gene clusters on chromosome 19, which were selectively hypomethylated in SETDB1-KO cells.

**Conclusions:**

SETDB1 collectively and specifically represses a substantial fraction of the C2H2-ZFP gene family. Through the en-bloc silencing of a set of ZFP genes, SETDB1 may help establish a panel of ZFP proteins that are expressed cell-type specifically and thereby can serve as signature proteins for cellular identity.

**Supplementary Information:**

The online version contains supplementary material available at 10.1007/s11033-024-09703-2.

## Introduction

SETDB1 (SET domain bifurcated-1) is a histone methyltransferase that catalyzes the transfer of methyl groups to histone H3 at lysine 9 (H3K9), resulting in the formation of HP1-mediated facultative heterochromatin [1–4] and transcriptional repression of genes, and thus plays an important role in the epigenetic regulation of gene expression. Because of the inability of SETDB1 to choose target loci, it depends on numerous transcription factors (TFs), including ERG [5]), KAP1 [6], SIN3 [7], PML [8, 9], SP3 [10], ATF7IP [11–14], HUSH complex [11], RESF1 [15], and others. Guided by these transcription factors, SETDB1 silences a range of genes, depending on the cellular context, that are involved in nervous system development, cell division, proliferation, immune cell regulation, etc. Dysregulation in SETDB1 expression is associated with tumorigenesis, neuropsychiatric and genetic disorders, as well as cardiovascular and gastrointestinal ailments [16]. Given SETDB1’s extensive cellular impacts, its significance in cell homeostasis is unparalleled.

SETDB1 is involved in DNA methylation by interacting with DNMTs at target sites. For instance, DNMTs and SETDB1 cooperate with the transcription factor MAX to repress developmentally regulated genes in embryonic stem cells through DNA methylation [17]. At the *TP53BP2* gene promoter in HeLa cells and the *RASSF1A* gene promoter in MDAMB-231 breast cancer cells, SETDB1 interacts with DNMT3A, but not with the maintenance methyltransferase DNMT1 [18]. In *Drosophila*, dSetdb1 recruits Dnmt2 and Su(var)205 to mediate DNA methylation [19]. These studies provide evidence indicating that SETDB1 collaborates with DNMTs in transcriptional repression. However, whole-genome analysis in mouse embryonic stem cells exhibited little overlap between de-repressed genes in *Setdb1*-null embryonic stem cells and DNMT1/3A/3B triple knockout (KO) cells [20]. Depletion of SETDB1 induces aberrant CTCF binding, leading to altered transcription independent of DNA methylation [21]. In addition, SETDB1 is necessary for H3K9me3 marking and silencing of endogenous retrovirus (ERV) subfamilies in mouse embryonic stem cells; however, the overall DNA methylation level at these ERVs is unaltered or only marginally decreased in *Setdb1* KO cells [22]. Furthermore, these retroelements have normal H3K9me3 levels in DNMT triple KO embryonic stem cells. These observations suggest that SETDB1 operates autonomously of DNA methylation in a region- and cell-specific manner and that only selective genes and loci may be affected by both SETDB1-mediated H3K9me3 and DNA methylation.

The existence of specific DNA-binding motifs that allow transcription factors (TFs) to bind to target gene promoters is crucial for TF function [23]. One of the most prevalent DNA-binding motifs in eukaryotic TFs is the zinc finger (ZNF), and zinc finger proteins (ZFPs) are the largest TF family. Based on the type and spacing of their zinc-chelating residues, several kinds of ZNF domains have been identified and characterized [24]. The canonical ZNF motif, C2H2-like finger (also known as Kruppel-like) which consists of 28–30 amino acid residues, is stabilized by a zinc ion coordinated by four highly conserved residues, two cysteines and two histidines [25]. Each ZNF motif is thought to bind an adjacent three-nucleotide subsequence, and each C2H2-ZNF domain can be tailored to a range of three base-pair (3 bp) targets [26]. Most C2H2-ZFPs (ZFPs with C2H2 domains) have other conserved domains that contribute to protein function. The Kruppel-associated box (KRAB) domain, for instance, interacts with corepressor proteins such as Krueppel-associated protein-1 (KAP1) to restrict the transcription of relevant genes [27]. With these regulatory functions, ZFPs are engaged in controlling critical physiological and pathological processes like development, differentiation, metabolism, apoptosis, and cancer [28]. The involvement of ZFPs in cancer biology is particularly noteworthy because ZFP expression is either increased or decreased in tumor samples and numerous ZFPs appear to play tumor-type-specific roles [29]. Given the specificity of various ZFPs in terms of function and expression for specific tumors, it may be desirable to employ this class of proteins as prognostic indicators [29].

This study analyzes SETDB1-KO human near-haploid (HAP1) cells through a linked analysis of the methylome and transcriptome, intending to explore genes and loci that are controlled by SETDB1-involved DNA methylation. The study found that DNA methylation was reduced in hundreds of C2H2-ZFP family genes and that the reduced DNA methylation in SETDB1-KO cells was strongly associated with higher expression of the corresponding C2H2-ZFP genes. The results indicate that SETDB1 collectively and specifically represses a substantial fraction of the C2H2-ZFP gene family in HAP1 cells. This repression may contribute to the selection of cell type-specific C2H2-ZFP genes that are required for maintaining cellular identities and fates.

## Materials and methods

### Cell culture and reverse transcription-polymerase chain reaction (RT-PCR)

Wild-type (WT) and SETDB1 knockout (KO) near-haploid human HAP1 cell line (C631, Horizon Discovery) were cultured in Iscove’s Modified Dulbecco’s Medium (IMDM; Gibco, Thermo Fisher Scientific) supplemented with 100 U/ml penicillin/streptomycin (Gibco, Thermo Fisher Scientific) and 10% heat-inactivated fetal bovine serum (FBS; HyClone) at 37 °C in 5% CO2. Cells were split 1:4 every two days using trypsin (Gibco, Thermo Fisher Scientific). For RT-PCR, total RNAs were extracted from HAP1 cells using RNeasy Plus Mini Kit (Qiagen) according to the manufacturer’s instructions. Ten µg of total RNA was treated with 10 U of recombinant DNase I (Takara) for 30 min at 37 °C, followed by purification using ethanol precipitation. Two µg of DNase I-treated total RNA was reverse transcribed using 50 pmol of random hexamers and 200 U of SuperScript III Reverse Transcriptase (Thermo Fisher Scientific). A quantitative real-time PCR (qPCR) was performed with 10 ng of cDNA and 2X SYBR Green Fast PCR Master Mix (Applied Biosystems) on QuantStudio 3 Real-Time PCR System (Applied Biosystems). For primer information, refer to Supplementary_datafile_1. Ct values of target genes were normalized by those of GAPDH transcripts using the –ΔΔCt method to calculate fold change.

### Reduced representation bisulfite sequencing (RRBS)

An in-house designed RRBS approach molecularly indexed and sequenced bisulfite-converted *Msp*I-restricted genomic segments (Keyomics, Daejeon, Korea) [30]. Two adaptors were used: Adaptor-A with a molecular barcode and R1 primer site, and Adaptor-B with an R2 primer site, both featuring bisulfite-resistant cytosines. Genomic DNA (100ng) underwent Zymo cleanup, adaptor ligation at 25 °C for 30 min with NEB Blunt/TA Ligase, followed by filling in using Tag DNA polymerase at 72 °C, substituting dCTP with methyl-dCTP. After bisulfite treatment using EZ DNA Methylation-Gold™ Kit, the fragments were PCR-amplified (10 cycles at 95 °C for 30 s, 60 °C for 30 s, and 72 °C for 30 s) using primers P1, P2, idxP1, and idxP2. The libraries were sequenced on an Illumina Hiseq platform with 151 bp paired-end reads.

### RNA sequencing (RNA-seq)

A detailed procedure for RNA-seq was described elsewhere [31]. Briefly, poly-A RNAs from 1 µg total RNA were isolated using the Dynabeads mRNA DIRECT kit (Thermo), DNase I-treated (Sigma) at 37 °C for 30 min, and fragmented at 94 °C for 15 min. cDNA synthesis involved ProtoScript II Reverse Transcriptase for the first strand and Second Strand Synthesis Enzyme Mix for the second, both included in the kit. Double-stranded DNAs were end-repaired using NEBNext End Prep Enzyme Mix at 20 °C for 30 min, then 65 °C for 30 min, ligated to NEBNext Adaptor with Blunt/TA Ligase Master Mix (NEB) at 20 °C for 15 min. Libraries were PCR-enriched (12–15 cycles) with universal and index primers using 2x Phusion High-Fidelity PCR Master Mix with HF Buffer (Thermo), quantified with NEBNext Library Quant Kit for Illumina, pooled, and sequenced on the Illumina HiSeqX system (2 × 100 bp).

### Production of CRISPR-mediated DNMT3A knockdown HAP1 cells

Oligonucleotides encoding DNMT3A-specific sgRNAs, 5’-CACCGGCATGATGCGCGGCCCAAGG-3’ for sgDNMT3A-1 and 5’-CACCGGGACATCTCGCGATTTCTCG-3’ for sgDNMT3A-2, were annealed with their complementary pairs (100 pmol each), phosphorylated with T4 kinase (NEB) at 37 °C for 30 min, and ligated into pSpCas9(BB)-2 A-Puro (PX459; Addgene) using T4 DNA ligase (NEB) for 2 h at 16 °C to produce PX459-sgDNMT3As. These plasmids were transfected into control HAP1 cells using LipofectaminTM 3000 (Thermo), and 48 h later, cells were analyzed by qPCR. Efficient CRISPR targeting was verified by observing reduced PCR amplicon sizes from the target locus.

### DNA and H3K9 methylation analysis of ZFP gene clusters in chromosome 19

Using the M-values, methylation differences at promoters (TSS ± 3 kb) of chr19 ZFP genes were examined. First, β-values were converted to M-values, and differentially methylated CpGs (DMCs, p-value < 0.0001, fold-change ≥ 2) between SETDB1-KO and WT cells were identified by LIMMA (v3.54.0). Next, whole RRBS CpGs mapped on chr19 were plotted along the chromosome length, and DMCs found in the promoters of chr19 ZFP genes were colored in red by ggplot2 (v3.4.1) to visualize alterations of DNA methylation in the absence of SETDB1. For H3K9me3 analysis, the raw fastq files generated from H3K9me3 ChIP-seq were preprocessed using Trim_galore (v0.6.8dev) to remove low-quality reads, adapter contamination, and other artifacts. The preprocessed reads were then aligned to the human reference genome (hg38) using the Burrows-Wheeler Aligner (BWA v0.7.17-r1188) with default parameters. The resulting BAM files were sorted and indexed using SAMtools (v1.14). To quantify the H3K9me3 enrichment, the number of reads in 50 bp genomic bins was counted using BEDTools intersect (v2.29.2), and the counts were normalized using the ‘cpm’ function in edgeR (v3.40.1). To compare the H3K9me3 enrichment between SETDB1-KO and WT samples, we calculated the fold enrichment in each bin, and statistical significances were calculated by LIMMA (v3.54.0). To examine the differential H3K9me3 enrichments in the promoters (TSS ± 3 kb) of chr19 ZFP genes, bins with an FDR less than 0.05 were mapped to human chromosome 19 (hg38), and the bins that overlapped with the promoters of ZNF genes were colored in navy by ggplot2 (v3.4.1). The omics data used in this work, including the methylome, transcriptome, and H3K9me3 ChIP-seq, were also used in our most recent paper [32].

## Results

### Methylome of SETDB1 knonckout HAP1 cells

**Fig. 1:**
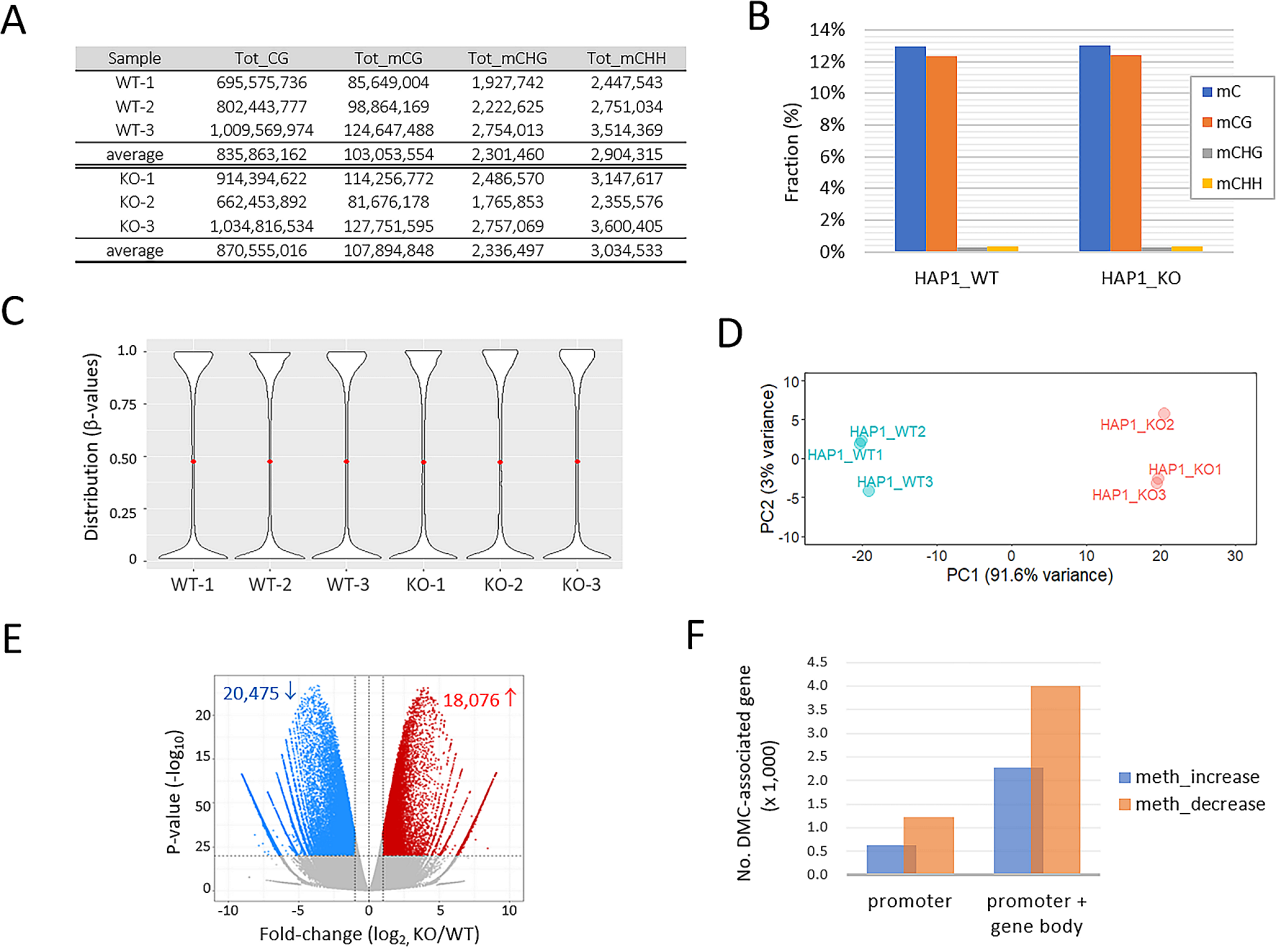
DNA methylome characteristics in SETDB1 knockout cells. (**A**) Quantitation of cytosine methylation at different sequence contexts such as CpG dinucleotides (mCG) and CpHpG (mCHG) and CpHpH (mCHH) trinucleotides in the RRBS reads of wild-type (WT) and SETDB1-KO (KO) HAP1 cells. Tot_CG counts the total number of CpG dinucleotides present in the RRBS reads. (**B**) Percentages of cytosine methylations at different sequence contexts. (**C**) Distribution of CpG methylation ratios (β-value). Red dots indicate the mean methylation. (**D**) Principal component analysis (PCA) using β-values. (**E**) Volcano plot showing differentially methylated CpGs (DMCs; KO/WT fold change > 2 and *p* < 0.0001), either in red (increased DMCs in KO) or in blue (decreased in KO). (**F**) Counts of DMCs found in promoters alone or in both the promoters and gene bodies

To look at changes in global DNA methylation in the absence of SETDB1, we used SETDB1-KO HAP1 cells. This cell line has a 14-bp deletion in exon-3 of the *SETDB1* gene and the depletion of the SETDB1 protein was confirmed by Western blotting (Supplementary Fig. S1A). We also observed a reduction in H3K9me3 levels in the SETDB1-KO cells (~ 66% of the control cells) and increased expressions in endogenous retrovirus (ERV) sequences, as known previously [22] (Supplementary Fig. S1B and **S1C**). These results confirmed the functional loss of SETDB1 in this cell line. We performed reduced-representation bisulfite sequencing (RRBS) [32]. Approximately 32 million reads per sample (*n* = 3 per group) were acquired (see Supplementary Fig. S2A and **S2B** for mapping statistics). About 850 million CpG sites contained in the collected *Msp*I fragments were analyzed and the proportion of methylated CpGs was ~ 12% in both WT and SETDB1-KO cells (Fig. 1A). 95% of cytosine methylation occurred in the CG context, while CHG and CHH methylations accounted for only 5% in both cells (Fig. 1B). Supplementary Fig. S2C depicts the quantitative profile of RRBS-captured MspI fragments to the number of intrinsic CpGs in those fragments is shown in, showing the prevalence of MspI fragments containing 2–7 CpGs as well as a proportional quantitative representation of the reads aligned to them. The minimum permissible depth to support methylation ratios (β-values, the fraction of methylation at a specific CpG site) was set at ten aligned reads, which accounted for a substantial proportion (> 80%) of each CpG site.

### Hypomethylation is predominant in genic regions of SETDB1-KO cells

Although the overall distribution of β-values was similar (Fig. 1C), PCA revealed a substantial difference between wild-type (WT) and SETDB1-KO cells (Fig. 1D). In the SETDB1-KO cells, 20,475 CpGs showed decreased methylation (hypomethylation) and 18,076 showed increased methylation (hypermethylation) with fold-change (FC) > 2 and p-value < 0.0001 (Fig. 1E and see also Supplementary_datafile_1). These differentially methylated CpG (DMC) groups were associated with 4,001 and 2,267 genes, respectively, in either promoter (transcription start site (TSS) ± 1 kb) or gene body (Fig. 1F). Out of the 1,863 DMCs found in gene promoter regions, 1,231 were hypomethylated and 632 were hypermethylated, indicating that two-thirds of the promoter DMCs were hypomethylated. The finding indicates that in SETDB1-KO cells, methylation changes in genic areas are more likely to be hypomethylation rather than hypermethylation. DMCs found in promoter regions were labeled as pDMCs (promoter DMCs) for ease of identification, while those located in either gene-body or promoter regions were collectively labeled as pgDMCs (promoter or gene-body DMCs).

### SETDB1 deletion results in hypomethylation of a substantial number of C2H2-ZFP genes

**Fig. 2:**
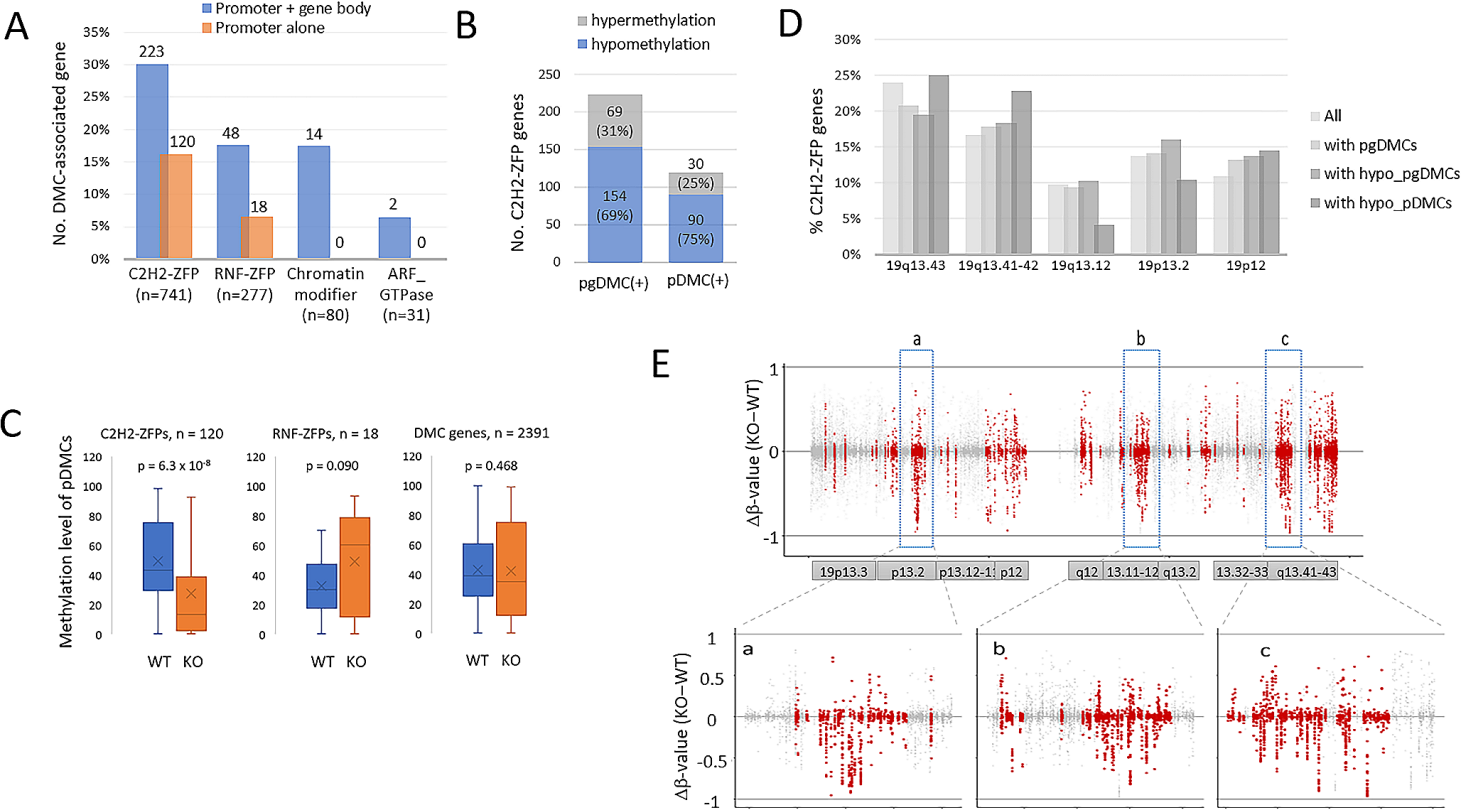
Enrichment of differentially methylated cytosines at the ZFP gene loci in SETDB1-KO cells. (**A**) Differentially methylated CpGs (DMCs) enriched in C2H2-ZFP family genes. Gene-associated DMCs (pgDMSs) are counted in the C2H2-ZFP (*n* = 741), RNF-ZFP (*n* = 277), chromatin-modifier (*n* = 80), and ARF-GTPase gene families (*n* = 31). (**B**) Fractions of C2H2-ZFP genes with hypermethylated DMCs or hypomethylated DMCs. DMC-containing ZFP genes are divided into those with DMCs in their promoter (pDMCs; transcription start site ± 1 kb; *n* = 120) only and genic regions (pgDMCs; promoter + gene body; *n* = 223). (**C**) Comparison of methylation levels of pDMCs in the C2H2-ZFP genes (*n* = 120), RNF-ZFP genes (right, *n* = 18), or entire genes (*n* = 2,391) between WT and SETDB1-KO cells. A two-sample t-test was used to determine statistical differences. (**D**) Distribution of C2H2-ZFP genes across the clusters in chromosome 19. The x-axis indicates the chromosomal locations of the major ZFP gene clusters. (**E**) β-value difference (KO – WT) for the 9,177 CpGs present in the promoters (TSS ± 3 kb, red) of the C2H2-ZFP genes clustered in chromosome 19. Grey dots denote whole RRBS CpGs in chromosome 19. There are 279 C2H2-ZFP genes in the chromosome-19 clusters and 254 have our RRBS CpG data. Boxed areas, a-c, are enlarged below

During our analysis of DMC-associated genes, we found a plethora of C2H2-ZFP genes. According to the HUGO Gene Nomenclature Committee (HGNC, https://www.genenames.org) website, there are 749 C2H2-ZFP genes, and 223 of them possessed pgDMCs; among these, 120 genes possessed pDMCs in their promoters (Fig. 2A and Supplementary_datafile_1). The proportion of C2H2-ZFP genes with pDMCs was notably higher (16.2%) than that of RING-finger domain ZFPs (RNF-ZFPs; 6.5%, *n* = 277) and other randomly selected gene families. In SETDB1-KO cells, C2H2-ZFP genes showed 69% and 75% net hypomethylation for pgDMCs and pDMCs, respectively (Fig. 2B). In the same context, the mean methylation levels of the pDMCs in the C2H2-ZFP genes were significantly reduced in SETDB1-KO cells. However, the levels of the pDMCs in the RNF-ZFP genes and the entire DMC-associated genes remained unchanged (Fig. 2C). This result indicates that the pDMCs in the C2H2-ZFP genes tend to be hypomethylated in SETDB1-deleted HAP1 cells. C2H2-ZFPs will be referred to as ZFPs in the following unless otherwise noted.

These ZFP genes are not localized but spread across human chromosomes. There are as many as 279 genes found in multiple clusters on chromosome 19 (chr19) alone [33]. Of these genes, 87 had pgDMCs that were hypomethylated in SETDB1-KO cells. We ruled out the possibility of SETDB1 affecting ZFP genes occurring on a cluster basis since the genes were spread out among the chr19 clusters (Fig. 2D). When evaluating the difference in β-values (ρβ = β_KO_ − β_WT_) for all 9,177 CpGs present in the chr19 ZFP gene promoters (TSS ± 3 kb), SETDB1-KO cells showed a significant tendency towards hypomethylation (Fig. 2E).

### Hypomethylation of ZFP genes is strongly correlated with their overexpression

**Fig. 3:**
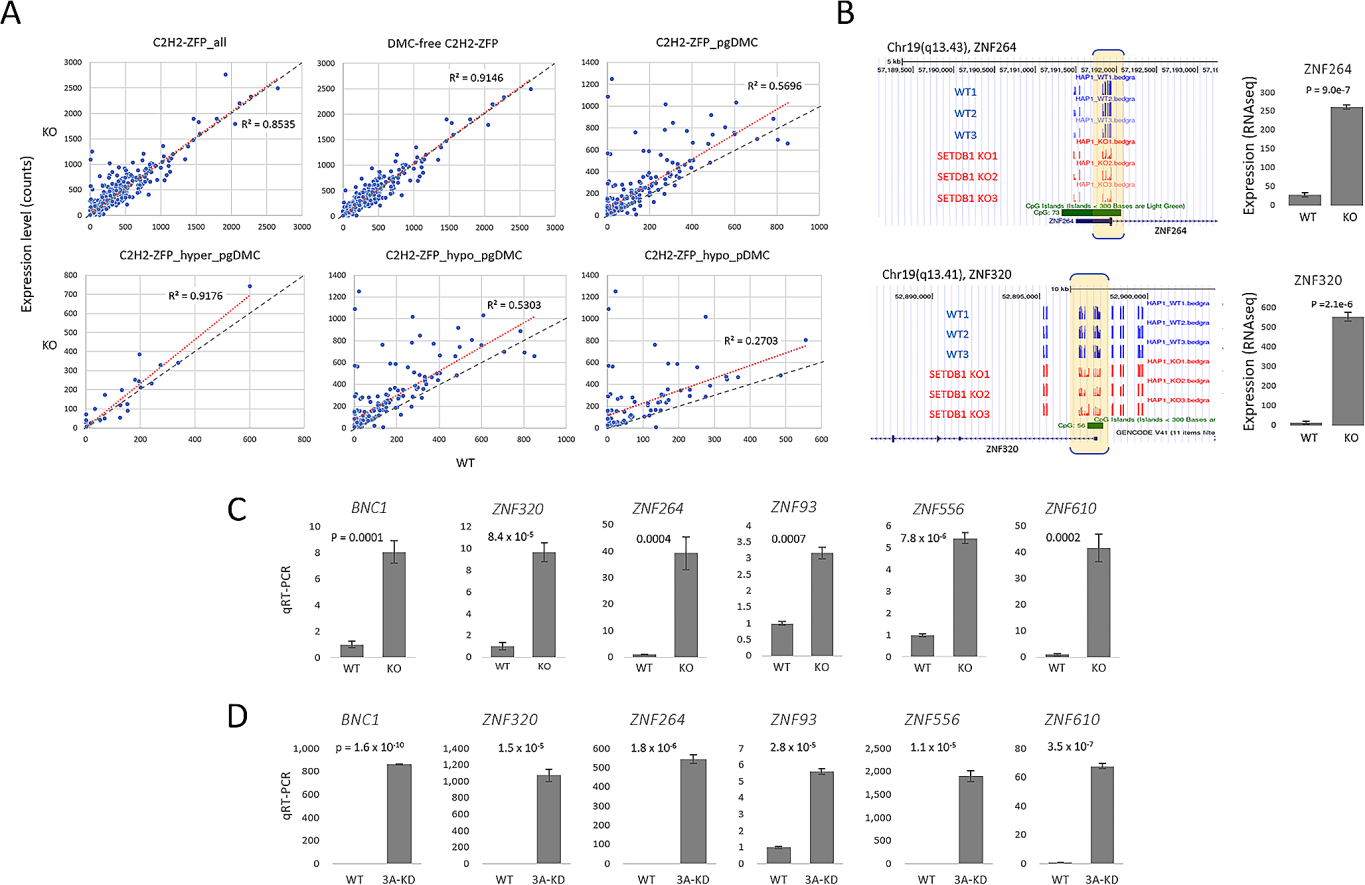
Upregulation of C2H2-ZFP genes in RNA-seq of SETDB1-KO cells. (**A**) Overexpression of ZFP genes with hypomethylated pDMCs in SETDB1-KO cells. The red and gray lines depict trend and reference (slope = 1) lines, respectively. There is a similarity between the upper right and lower middle plots, but they are not identical; this is because the elements in the latter plot also belong to the former plot. A total of 698 C2H2-ZFP genes expressed in HAP1 cells are evaluated, with genes that are not expressed in both WT and SETDB1-KO cells eliminated. (**B**) Snapshots of DNA methylation at ZFP gene promoters on the genome browser. The differentially methylated region is shaded in yellow in the bracket. CpG islands are depicted by a thick green bar. The bar graphs on the right show the RNA-seq expression levels of corresponding genes in WT and SETDB1-KO HAP1 cells. (**C-D**) Validation of increased expression of various C2H2-ZFP genes with hypomethylated DMCs in their promoters in the SETDB1-KO HAP1 cells (**C**) and CRISPR-mediated DNMT3A-knockdown HAP1 cells (**D**) by quantitative real-time PCR (qPCR). The error bars represent the standard deviation. qPCR values are averages of at least six technical replicates. P-values are indicated (paired-sample t-test)

Our findings indicate that SETDB1 depletion causes hypomethylation in a large group of ZFP genes, which may affect the expression levels of corresponding ZFP genes. To test this possibility, we evaluated the expression levels of ZFP genes in the transcriptome of SETDB1-KO cells [32]. The scatter plots comparing SETDB1-KO vs. WT expression levels revealed a marked difference between DMC-associated ZFP genes and DMC-free ZFP genes (R^2^ = 0.9146 vs. 0.5696; Fig. 3A). The difference was particularly noticeable for ZFP genes associated with hypomethylated pgDMCs (R^2^ = 0.5303) and even more so for those associated with hypomethylated pDMCs (R^2^ = 0.2703). The analysis indicates a significant correlation between the hypomethylated DMCs and the corresponding ZFP genes’ transcriptional enhancements. Supplementary Fig. S3 displays the expression heatmaps for each ZFP gene subset. Figure 3B shows a typical hypomethylation event in the ZNF264 and ZNF320 genes in SETDB1-KO HAP1 cells, along with the increased transcriptions of the associated genes.

We investigated whether this tendency repeats in other cells. For this, we depleted SETDB1 in 293T cells (Supplementary Fig. S4A). As in SETDB1-KO HAP1 cells, the majority of ZFP genes showed an increased expression in SETDB1-KO 293T cells (79%, 563/715 genes, with no cut-off condition; Supplementary Fig. S4B). Moreover, there were 289 differentially expressed genes (*p* < 0.01 and FC > 2) that were shared by HAP1 and 293T SETDB1-KO cells, of which 70 genes were C2H2-ZFP family genes (24%; Supplementary_datafile_1). The result suggests the universality of SETDB1’s role in ZFP gene regulation. Quantitative real-time PCR confirmed the overexpression of these ZFP genes in SETDB1-KO HAP1 and 293T cells (Fig. 3C and Supplementary Fig. S4C, respectively).

Additionally, we examined whether the expression levels of the ZFP genes with DMCs could be influenced by the DNA methyltransferase (DNMT) proteins. We chose DNMT3A based on its known interaction with SETDB1 (see Discussion). The results of CRISPR/Cas9-mediated knockdown of the *DNMT3A* gene followed by qPCR showed that a significant rise in ZFP gene expression levels was observed in HAP1 cells (Fig. 3D), similar to the observations in the *SETDB1* KO HAP1 and 293T cells. This finding suggests that DNMT3A cooperates with SETDB1 to install DNA methylation in the ZFP gene promoters. Meanwhile, to search for candidate transcription factors that could preferentially bind the promoters of the ZFP genes with pDMCs compared to the other ZFPs, we analyzed transcription factor binding sites (TFBS) across the two gene sets but we failed to find any significant difference in them (Supplementary Fig. S5).

### DNA methylation change is linked to H3K9 methylation change in the ZFP genes

**Fig. 4:**
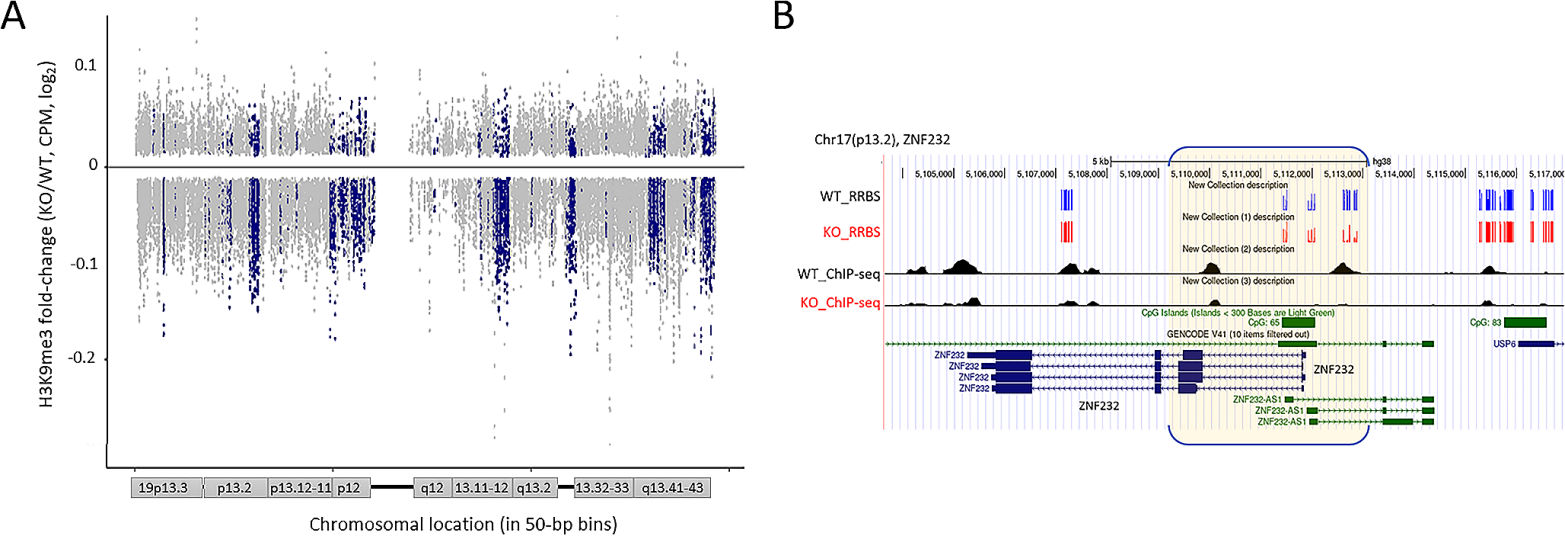
Reduction of H3K9me3 at the C2H2-ZFP gene promoters in SETDB1 KO cells. (**A**) H3K9me3 decline at promoters of chr19 ZNF genes in SETDB1-KO cells. Fold differences (KO/WT, log_2_ scale) of H3K9me3 ChIP-seq results were displayed across chromosome 19 (in 50-bp bins; x-axis). Blue dots represent bins that coincide with ZFP gene promoters (TSS ± 3 kb), while gray dots indicate bins of neighboring genes. CPM indicates counts per million. (**B**) Genome browser snapshot for H3K9me3 (ChIP-seq) and CpG methylation (RRBS) at the ZFP232 gene promoter (highlighted by yellow brackets). The promoter region overlaps with a CpG island, depicted by a green bar

We performed chromatin immunoprecipitation sequencing (ChIP-seq) for H3K9 trimethylation (H3K9me3), which is catalyzed by SETDB1 and may crosstalk with DNA methylation [34–37], to assess whether the SETDB1-induced DNA hypomethylation in the ZFP gene loci was associated with changes in H3K9me3. We observed a decrease in H3K9me3 levels at promoters within the ZFP gene-rich clusters on chromosome 19 in SETDB1-KO cells (Fig. 4A), reflecting the DNA methylation pattern in the matching clusters (Fig. 2E). Notably, although the reduction of H3K9me3 was extensive across chr19, the decrease was more pronounced in the ZFP gene-rich, hypomethylated regions compared to the neighboring regions. Figure 4B shows a snapshot of DNA and H3K9 methylation at the *ZNF232* gene locus on the Genome Browser; H3K9me3 levels in the promoters and gene bodies were decreased in the SETDB1-KO cells.

## Discussion

In this study, we found that many ZFP genes undergo DNA demethylation and elevated expression without SETDB1. The upregulation of many (up to one hundred) ZFP genes in SETDB1-KO cells may reflect the derepression of normally suppressed genes or significant increases in existing expressions, distorting the cell type-specific profile of ZFP proteins and ultimately disrupting functional equilibrium and cellular homeostasis. The resulting pleiotropic effects caused by SETDB1 depletion are so vast and complicated that they make it difficult to understand and predict the subsequent molecular events in SETDB1 KO cells. SETDB1 and ZFPs are known to collaborate to suppress genomic regions including endogenous retroviruses (ERVs) [2, 22, 38, 39]. Krüppel-associated box domain ZFPs (KRAB-ZFPs, KZFPs), which are the largest subset of the C2H2-ZFP family and constitute one of the largest families of transcriptional regulators [40, 41], recognize ERV elements in a sequence-specific manner and recruit KAP1 (also known as TRIM28). KAP1 then acts as a scaffold for the formation of the heterochromatin-inducing machinery, which includes SETDB1, HP1, and other nucleosome-remodeling proteins, to silence the target regions by depositing H3K9me3 [42–44]. DNA methylation is also mediated by the KZFP-KAP1-SETDB1 complex, but only occasionally and in cell type- and locus-specific manners (refer to Introduction). Collectively, it is assumed that the DNA methylation of a subset of ZFP genes is mediated under the operation of the KZFP-KAP1-SETDB1 complex and primarily depends on SETDB1. This mechanism is notable because the targets of SETDB1-related, DNA methylation-involved repression are the ZFP genes themselves that confer target specificity to SETDB1. We envision a regulatory model in which a cell-specific, master ZFP protein(s) attracts SETDB1 (probably via the linker KAP1) to repress subordinate ZFP genes that should be silenced for the maintenance of cellular identity. Supporting this model, ZNF274 in K562 cells has been shown to attract SETDB1 to multiple ZFP gene loci [45], although it remained elusive if the association of ZNF274 can cause transcriptional repression of these ZFP gene loci.

The chr19 ZFP genes were previously found to have prominent SETDB1 and H3K9me3 peaks [45, 46]. In those studies, SETDB1 and H3K9me3 peaks were found to localize to the 3’ end of the ZFP genes in K562 [45]. Similarly, we detected a similar localization of H3K9me3 peaks at the 3’ end in our analysis of the public HEK293-derived GSE175195 dataset (data not shown). The results are intriguing but also raise several questions, such as the epigenetic implications of SETDB1 association at the 3’-end of ZFP gene loci rather than in the promoter region, and what the SETDB1 binding to the 3’ends of ZFP genes might mean for gene expression. Further research is needed to understand the potential epigenetic implications of this unusual SETDB1 3’-end association and its possible effects on gene expression. Meanwhile, no specific pattern of DNA methylation at the 3’ end of ZFP genes was observed in HAP1 and 293T cells (data not shown), suggesting that the deposition of H3K9me3 and 5-methylcytosines at the 3’-end of ZFP genes are not related in these cells.

The coordinated regulation of chr19 ZFP genes has also been observed in cancer and aging studies. However, in contrast to our methylomes showing overall hypomethylation of these ZFP genes in the SETDB1-KO cells, the methylomes from oropharyngeal squamous cell carcinoma [47] and the peripheral mononuclear cells (PBMCs) collected from the aging population [48] showed enrichment of hypermethylation across the ZFP gene clusters. These results raise the possibility of an increase in SETDB1 activity with age and cancer. However, SETDB1 expression is relatively stable across tissues and ages [31, 49–51], suggesting another mechanism that spreads and intensifies DNA methylation in chr19 ZFP genes in cancer and aging. Meanwhile, we recently observed that SETDB1 controls the largest miRNA cluster in chr19 (q13.42) in HAP1 cells [32]. Since this miRNA cluster is very close to one of the ZFP gene clusters, and the dozens of miRNA genes located there are all significantly upregulated in SETDB1-KO HAP1 cells, it will be interesting to see if the two different classes of genes in the nearby chromosomal region are subject to coordinated regulation by SETDB1 and DNA methylation.

SETDB1 may be involved in DNA methylation via transcriptional control of DNMTs or interaction with DNMTs at target loci. In support of this, it is known that SETDB1 and DNMTs have a direct physical connection and functional association. DNMTs and SETDB1 cooperate with the transcription factor MAX to repress developmentally regulated genes in embryonic stem cells through DNA methylation [17]. At the *TP53BP2* gene promoter in HeLa cells and the *RASSF1A* gene promoter in MDAMB-231 breast cancer cells, SETDB1 interacts preferentially with the de novo DNA methyltransferase DNMT3A, but not with the maintenance methyltransferase DNMT1 [18]. The latter result seems to be close to ours because, as seen in the genome browser snapshots showing localized demethylation in Fig. 3B, the DNA methylation changes were so localized that the DNMT1, with its typical role as a maintenance methyltransferase, is an unlikely primary culprit for the SETDB1-mediated methylation changes. Moreover, the ZFP genes upregulated in the SERTDB1-KO cells were also upregulated by CRISPR-mediated DNMT3A knockdown in HAP1 cells (Fig. 3C and D), suggesting a collaboration between SETDB1 and DNMT3A for ZFP repression via DNA methylation. These observations lead us to speculate that the loss of interaction between SETDB1 and DNMT3A causes local demethylation in ZFP gene promoters in SETDB1-KO cells. In this way, SETDB1, in cooperation with DNMT proteins, appears to play a crucial role in certain genes and loci where the preservation of DNA methylation is essential.

We observed that while the DNA methylation changes were more exclusive to the ZFP gene clusters, the H3K9me changes were rather widespread across chr19 (Fig. 4A). In distantly related organisms, the methylation of histone H3 at lysine K9 (H3K9) works in tandem with DNA methylation to maintain the silencing of genes and repetitive elements, and it tightly controls the DNA methylation pattern [35]. In mammals, the relationship between the two epigenetic marks is less clear, as H3K9-specific histone methyltransferases (HMTases) appear to act either in conjunction with or independently of DNA methylation, depending on the target sequence [18, 20]. All three groups of authentic H3K9-specific HMTases, SUV39H1/SUV39H2, G9A/GLP, and SETDB1, have been observed to affect DNA methylation at specific loci [22, 32, 36, 49]. In this study, we did not perform an exhaustive gene-to-gene investigation for the two marks because they are not directly comparable. From a technical perspective, RRBS may have an inbuilt limit in gathering genome-wide methylome data due to the specific restriction enzyme, *Msp*I, used in RRBS library generation, with methylation data largely biased toward the CGIs. The highly localized presence of RRBS-derived reads on CGIs results in a different genomic signature than the global presence of H3K9me3 ChIP-seq reads. Thus the H3K9me3 ChIP-seq profile, in which CGIs are usually minimally represented, and the RRBS methylation profile, in which CGIs are almost completely represented, are inherently different from the start and thus inappropriate for direct comparison.

## Conclusion

We found that SETDB1 represses a large subset of ZFP genes. In SETDB1-KO cells, this set of ZFP genes exhibited DNA hypomethylation and gene upregulation, features not seen in other random gene families. Through the en bloc silencing of a set of ZFPs, SETDB1 negatively selects ZFP proteins and the compensatory ZFPs may constitute a cell type-specific profile of ZFP proteins that can serve as markers for cellular identification. This is consistent with the observation that certain ZFPs are associated with specific cancers and may be used as prognostic indicators [29]. We imagine that this SETDB1-selected group of ZFPs, as the transcription factors associated with a specific cellular fate, may fulfill the cell’s genetic program to manifest the cell’s physiological and biochemical properties. As a cell undergoes differentiation and other cellular transitions, the composition of the ZFP expression panel changes, revealing a distinct signature at each stage. In some cases, malignancies and cells exposed to altered SETDB1 activity [50, 51] can transform into a composite population with panels of ZFPs of ambiguous identity.

## Electronic supplementary material

Below is the link to the electronic supplementary material.

Supplementary Material 1

Supplementary Material 2

## Data Availability

No datasets were generated or analysed during the current study.

## Abbreviations

CpG: CG dinucleotide
DEG: Differentially expressed gene
DMC: Differentially methylated CpGs
RRBS: Reduced-representation bisulfite sequencing
C2H2-ZFP: Cystine-2 histidine-2 amino-acid sequence motif-type zinc-finger protein
ZNF: Zinc finger
ZFP: Zinc-finger protein
TFBS: Transcription factor binding site
