# Supplementary material for: SETDB1 deletion causes DNA demethylation and upregulation of multiple zinc-finger genes
Source: Mol Biol Rep. 2024 Jun 21;51(1):778. doi: 10.1007/s11033-024-09703-2 (PMC11192681; doi:10.1007/s11033-024-09703-2)
Supplement: Supplementary file 2 — Supplementary Material 2 [file 11033_2024_9703_MOESM2_ESM.pptx]

## Slide 1
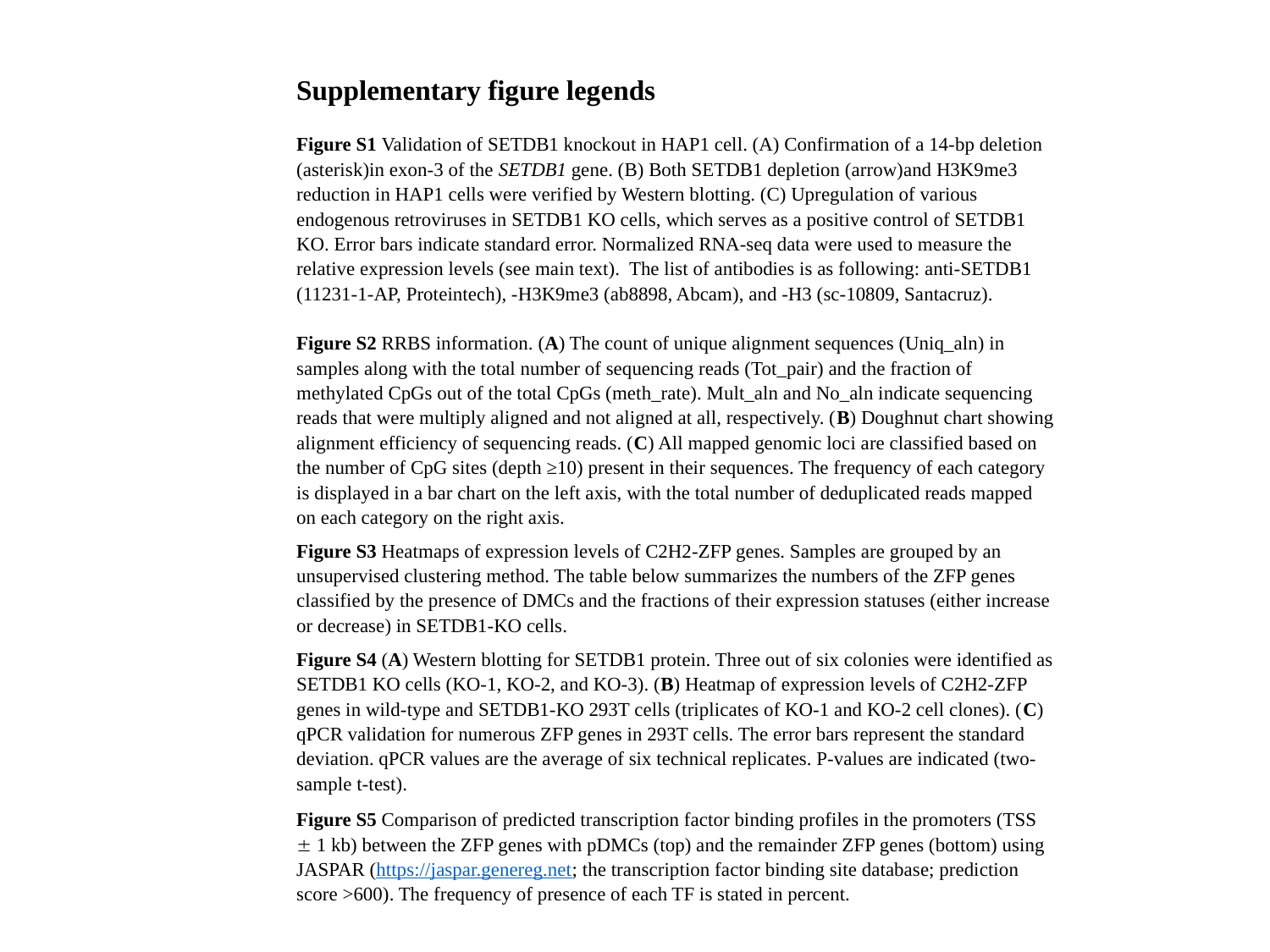

Supplementary figure legends
Figure S1 Validation of SETDB1 knockout in HAP1 cell. (A) Confirmation of a 14-bp deletion (asterisk)in exon-3 of the SETDB1 gene. (B) Both SETDB1 depletion (arrow)and H3K9me3 reduction in HAP1 cells were verified by Western blotting. (C) Upregulation of various endogenous retroviruses in SETDB1 KO cells, which serves as a positive control of SETDB1 KO. Error bars indicate standard error. Normalized RNA-seq data were used to measure the relative expression levels (see main text). The list of antibodies is as following: anti-SETDB1 (11231-1-AP, Proteintech), -H3K9me3 (ab8898, Abcam), and -H3 (sc-10809, Santacruz).
Figure S2 RRBS information. (A) The count of unique alignment sequences (Uniq_aln) in samples along with the total number of sequencing reads (Tot_pair) and the fraction of methylated CpGs out of the total CpGs (meth_rate). Mult_aln and No_aln indicate sequencing reads that were multiply aligned and not aligned at all, respectively. (B) Doughnut chart showing alignment efficiency of sequencing reads. (C) All mapped genomic loci are classified based on the number of CpG sites (depth ≥10) present in their sequences. The frequency of each category is displayed in a bar chart on the left axis, with the total number of deduplicated reads mapped on each category on the right axis.
Figure S3 Heatmaps of expression levels of C2H2-ZFP genes. Samples are grouped by an unsupervised clustering method. The table below summarizes the numbers of the ZFP genes classified by the presence of DMCs and the fractions of their expression statuses (either increase or decrease) in SETDB1-KO cells.
Figure S4 (A) Western blotting for SETDB1 protein. Three out of six colonies were identified as SETDB1 KO cells (KO-1, KO-2, and KO-3). (B) Heatmap of expression levels of C2H2-ZFP genes in wild-type and SETDB1-KO 293T cells (triplicates of KO-1 and KO-2 cell clones). (C) qPCR validation for numerous ZFP genes in 293T cells. The error bars represent the standard deviation. qPCR values are the average of six technical replicates. P-values are indicated (two-sample t-test).
Figure S5 Comparison of predicted transcription factor binding profiles in the promoters (TSS ± 1 kb) between the ZFP genes with pDMCs (top) and the remainder ZFP genes (bottom) using JASPAR (https://jaspar.genereg.net; the transcription factor binding site database; prediction score >600). The frequency of presence of each TF is stated in percent.

## Slide 2
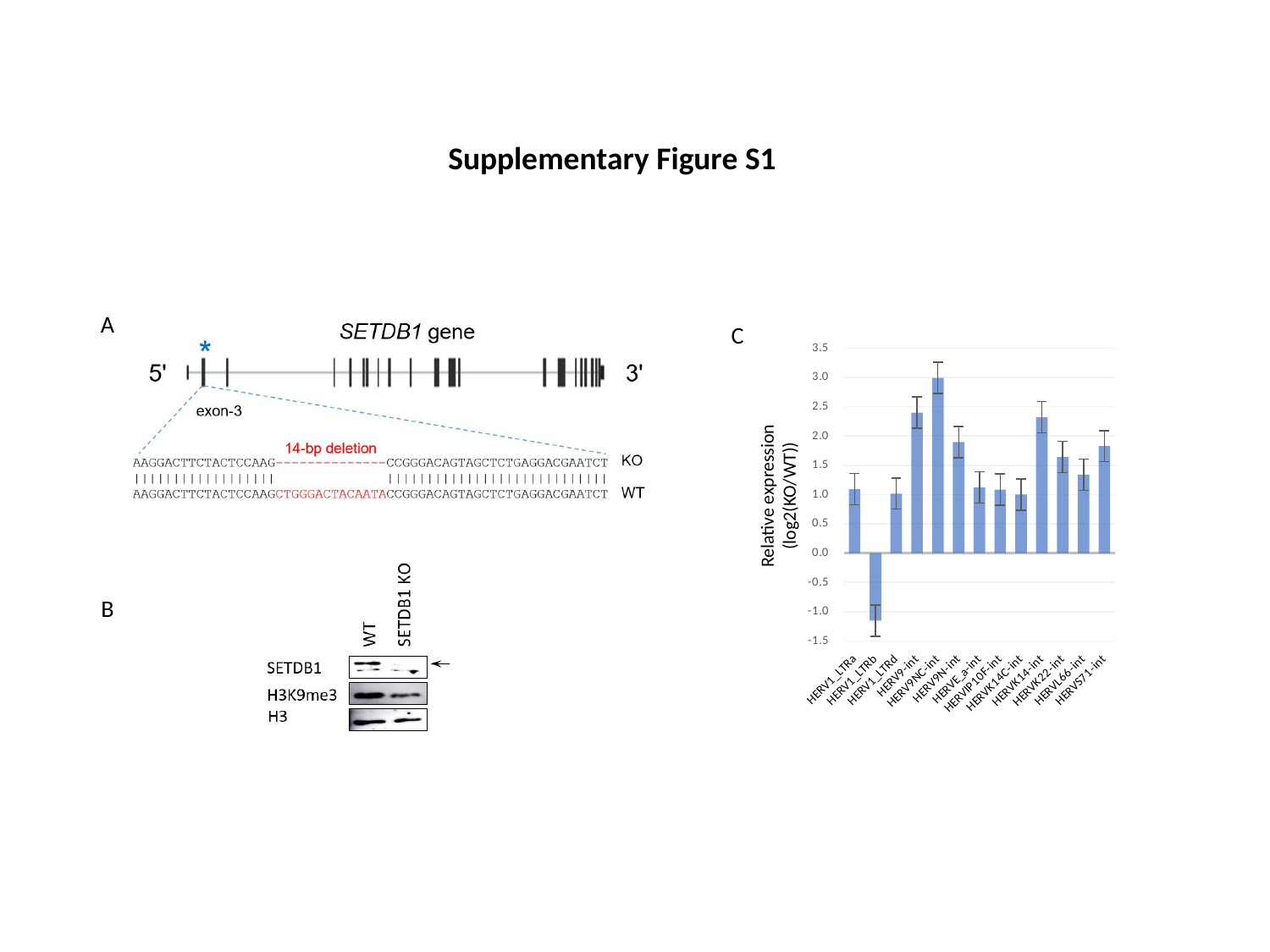

Supplementary Figure S1
A
C
### Chart
| Category | |
|---|---|
| HERV1_LTRa | 1.0921598557224856 |
| HERV1_LTRb | -1.1540007315469116 |
| HERV1_LTRd | 1.0159242698954007 |
| HERV9-int | 2.40015572239307 |
| HERV9NC-int | 2.9928406567823598 |
| HERV9N-int | 1.8966253415230565 |
| HERVE_a-int | 1.12233049690034 |
| HERVIP10F-int | 1.0830384657539376 |
| HERVK14C-int | 1.0003274148443821 |
| HERVK14-int | 2.3199098024495224 |
| HERVK22-int | 1.6431584234048038 |
| HERVL66-int | 1.3407639602026662 |
| HERVS71-int | 1.826526312479501 |Relative expression
(log2(KO/WT))
B

## Slide 3
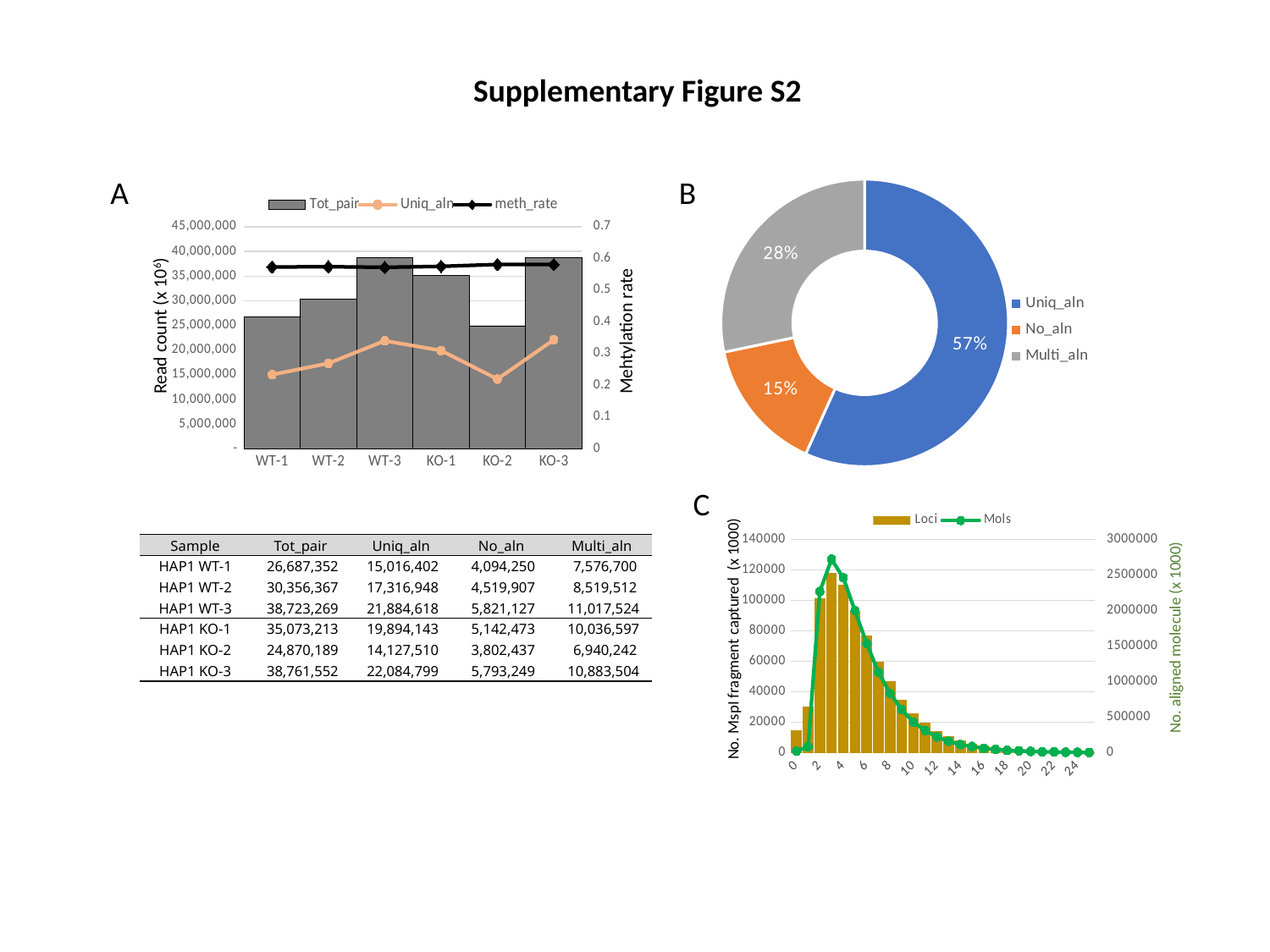

Supplementary Figure S2
A
B
### Chart
| Category | |
|---|---|
| Uniq_aln | 110324420.0 |
| No_aln | 29173443.0 |
| Multi_aln | 54974079.0 |
### Chart
| Category | Tot_pair | Uniq_aln | meth_rate |
|---|---|---|---|
| WT-1 | 26687352.0 | 15016402.0 | 0.573 |
| WT-2 | 30356367.0 | 17316948.0 | 0.574 |
| WT-3 | 38723269.0 | 21884618.0 | 0.572 |
| KO-1 | 35073213.0 | 19894143.0 | 0.575 |
| KO-2 | 24870189.0 | 14127510.0 | 0.581 |
| KO-3 | 38761552.0 | 22084799.0 | 0.581 |Read count (x 106)
Mehtylation rate
C
### Chart
| Category | Loci | Mols |
|---|---|---|
| 0 | 14886.0 | 25069.0 |
| 1 | 30552.0 | 86398.0 |
| 2 | 101439.0 | 2269836.0 |
| 3 | 118092.0 | 2723268.0 |
| 4 | 110396.0 | 2463159.0 |
| 5 | 93322.0 | 1999097.0 |
| 6 | 76953.0 | 1537774.0 |
| 7 | 59824.0 | 1135818.0 |
| 8 | 46903.0 | 837480.0 |
| 9 | 35072.0 | 607011.0 |
| 10 | 25950.0 | 431126.0 |
| 11 | 19829.0 | 315566.0 |
| 12 | 14279.0 | 223199.0 |
| 13 | 10752.0 | 162654.0 |
| 14 | 7990.0 | 117591.0 |
| 15 | 6196.0 | 89525.0 |
| 16 | 4776.0 | 63582.0 |
| 17 | 3688.0 | 49493.0 |
| 18 | 2874.0 | 35607.0 |
| 19 | 2239.0 | 28429.0 |
| 20 | 1738.0 | 21053.0 |
| 21 | 1442.0 | 15455.0 |
| 22 | 1197.0 | 12134.0 |
| 23 | 919.0 | 8758.0 |
| 24 | 757.0 | 7079.0 |
| 25 | 617.0 | 5234.0 |No. aligned molecule (x 1000)
No. MspI fragment captured (x 1000)
| Sample | Tot\_pair | Uniq\_aln | No\_aln | Multi\_aln |
| --- | --- | --- | --- | --- |
| HAP1 WT-1 | 26,687,352 | 15,016,402 | 4,094,250 | 7,576,700 |
| HAP1 WT-2 | 30,356,367 | 17,316,948 | 4,519,907 | 8,519,512 |
| HAP1 WT-3 | 38,723,269 | 21,884,618 | 5,821,127 | 11,017,524 |
| HAP1 KO-1 | 35,073,213 | 19,894,143 | 5,142,473 | 10,036,597 |
| HAP1 KO-2 | 24,870,189 | 14,127,510 | 3,802,437 | 6,940,242 |
| HAP1 KO-3 | 38,761,552 | 22,084,799 | 5,793,249 | 10,883,504 |

## Slide 4
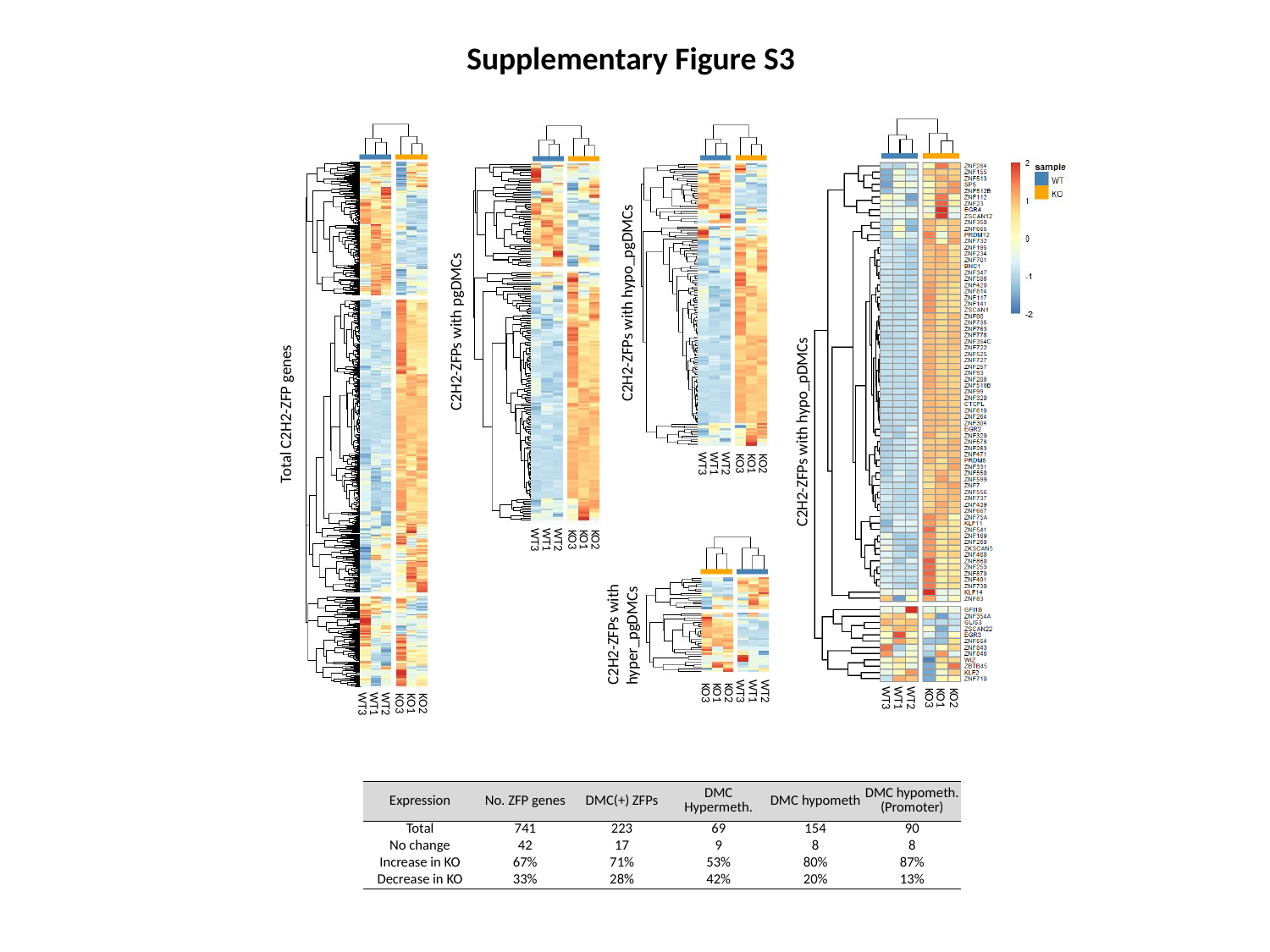

Supplementary Figure S3
WT3
WT1
WT2
KO3
KO1
KO2
C2H2-ZFPs with hypo_pDMCs
Total C2H2-ZFP genes
WT3
WT1
WT2
KO3
KO1
KO2
C2H2-ZFPs with pgDMCs
WT3
WT1
WT2
KO3
KO1
KO2
C2H2-ZFPs with hypo_pgDMCs
WT3
WT1
WT2
KO3
KO1
KO2
C2H2-ZFPs with hyper_pgDMCs
WT3
WT1
WT2
KO3
KO1
KO2
| Expression | No. ZFP genes | DMC(+) ZFPs | DMC Hypermeth. | DMC hypometh | DMC hypometh. (Promoter) |
| --- | --- | --- | --- | --- | --- |
| Total | 741 | 223 | 69 | 154 | 90 |
| No change | 42 | 17 | 9 | 8 | 8 |
| Increase in KO | 67% | 71% | 53% | 80% | 87% |
| Decrease in KO | 33% | 28% | 42% | 20% | 13% |

## Slide 5
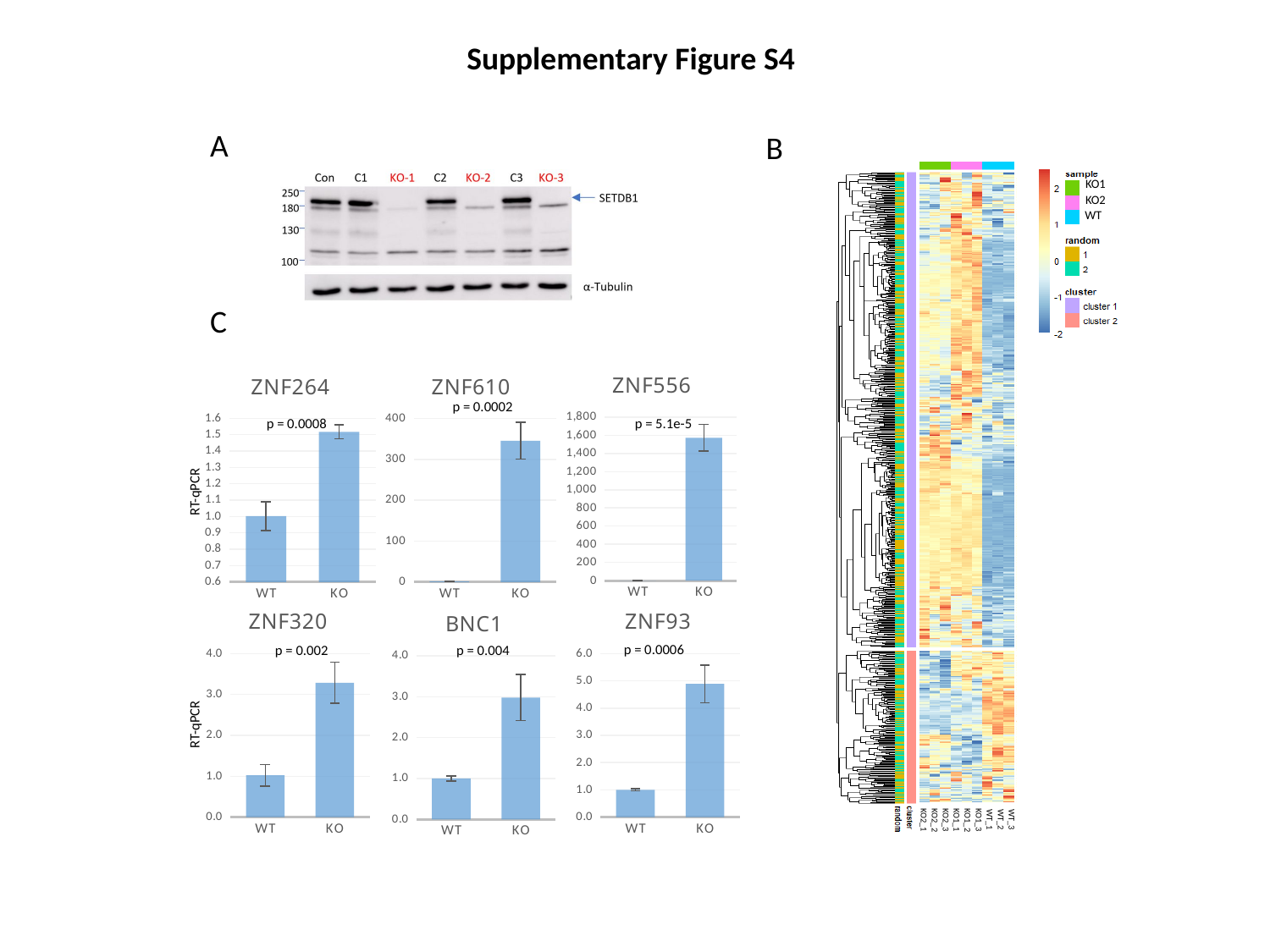

Supplementary Figure S4
A
B
KO1
KO2
WT
WT_1
WT_2
WT_3
KO2_1
KO2_3
KO1_1
KO1_3
KO2_2
KO1_2
C
### Chart:
| Category | ZNF556 |
|---|---|
| WT | 1.2937611599881544 |
| KO | 1572.0274508209059 |
### Chart:
| Category | ZNF264 |
|---|---|
| WT | 1.0025132099813856 |
| KO | 1.5165785106851544 |
### Chart:
| Category | ZNF610 |
|---|---|
| WT | 1.0000355287787588 |
| KO | 345.4754682747525 |p = 0.0002
p = 0.0008
p = 5.1e-5
RT-qPCR
### Chart:
| Category | ZNF320 |
|---|---|
| WT | 1.0228523756002201 |
| KO | 3.286438066578111 |
### Chart:
| Category | ZNF93 |
|---|---|
| WT | 1.0005296713504774 |
| KO | 4.885572520032125 |
### Chart:
| Category | BNC1 |
|---|---|
| WT | 1.0012763752223641 |
| KO | 2.9789500229333608 |p = 0.0006
p = 0.002
p = 0.004
RT-qPCR

## Slide 6
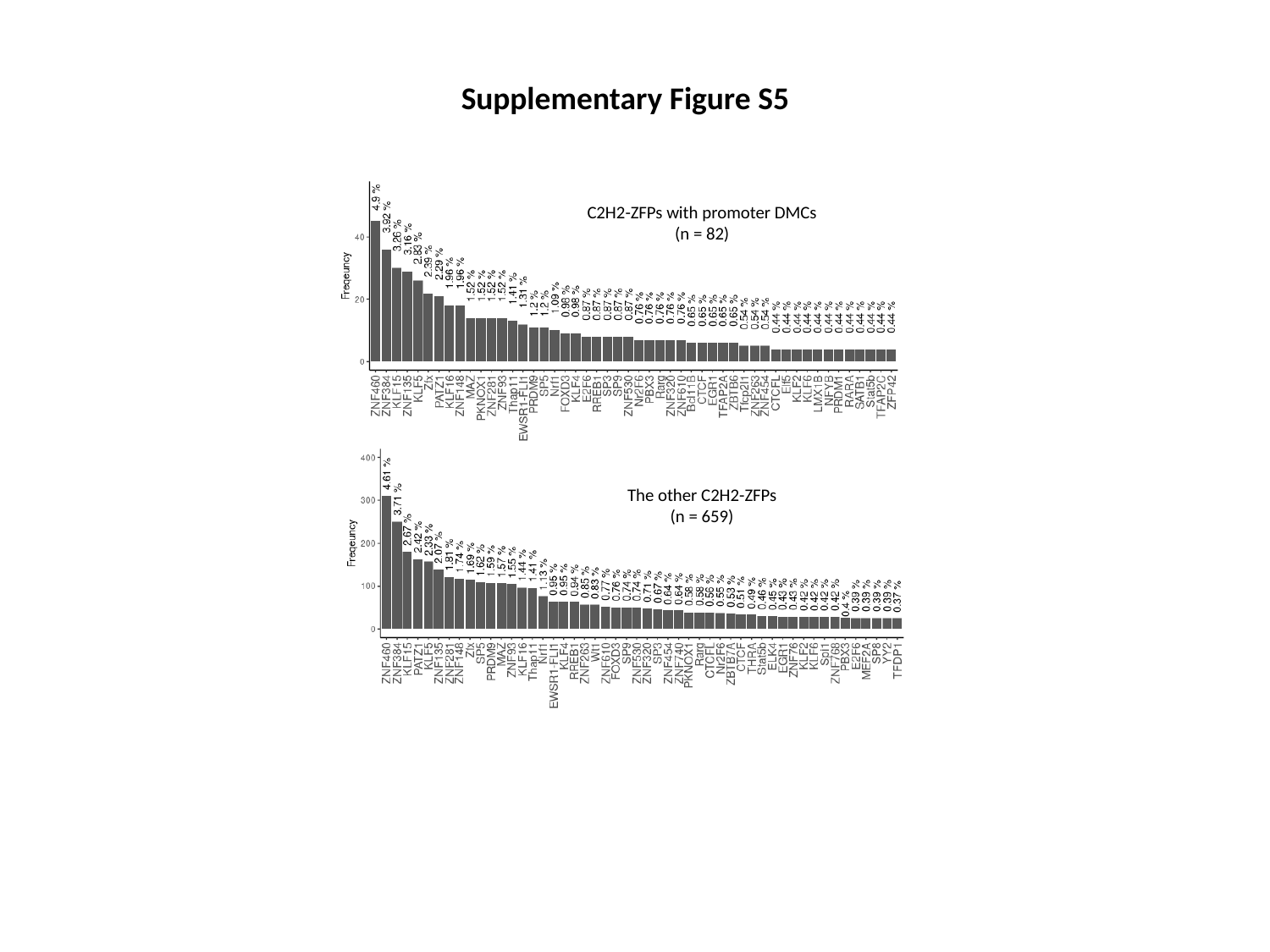

Supplementary Figure S5
C2H2-ZFPs with promoter DMCs
(n = 82)
The other C2H2-ZFPs
(n = 659)
